# Neutralization of SARS-CoV-2 (lineage B.1.1) by hyperimmune llama (*Lama glama*) serum in vero cell culture

**DOI:** 10.17843/rpmesp.2023.403.12509

**Published:** 2023-09-27

**Authors:** Verónica Yaniro, Silvia Capristano, Henri Bailon, Juan Lévano, Marco Galarza, David García, Omar Cáceres, Carlos Padilla, Harrison Montejo, Paquita García, Mary Celis, Silvia Seraylan, Yessica Garayar, Miryam Palomino

**Affiliations:** 1 National Referral Laboratory for Biotechnology and Molecular Biology, Centro Nacional de Salud Pública, Instituto Nacional de Salud, Lima, Peru. National Referral Laboratory for Biotechnology and Molecular Biology Centro Nacional de Salud Pública Instituto Nacional de Salud Lima Peru; 2 National Referral Laboratory for Viral Metaxenical Infections, Centro Nacional de Salud Pública, Instituto Nacional de Salud, Lima, Peru. National Referral Laboratory for Viral Metaxenical Infections Centro Nacional de Salud Pública Instituto Nacional de Salud Lima Peru; 3 Laboratorio de Referencia Nacional de Virus Respiratorios, Centro Nacional de Salud Pública, Instituto Nacional de Salud, Lima, Perú. Laboratorio de Referencia Nacional de Virus Respiratorios Centro Nacional de Salud Pública Instituto Nacional de Salud Lima Peru; 4 Centro Nacional de Producción de Biológicos, Instituto Nacional de Salud, Lima, Peru. Centro Nacional de Producción de Biológicos Instituto Nacional de Salud Lima Peru

**Keywords:** Coronavirus, SARS-CoV-2, Lama glama, Neutralization Tests, COVID-19 Serotherapy

## Abstract

**Objective.:**

To evaluate the serological antibody response of a llama (*Lama glama*) to SARS-CoV-2 (B.1.1 lineage) immunization and the neutralizing capacity of hyperimmune llama serum against SARS-CoV-2 virus (B.1.1 lineage) in Vero cells.

**Materials and methods.:**

A llama was immunized with inactivated SARS-CoV-2 (B.1.1 lineage). Serum samples were analyzed to evaluate the level of antibodies by ELISA, as well as reactivity to SARS-CoV-2 antigens by Western Blot. In addition, viral neutralization in cell cultures was assessed by the Plate Reduction Neutralization Test (PRNT).

**Results:**

. Seroreactivity increased in the immunized llama from week 4 onwards. Antibody titers were the highest after the seventh immunization booster. Western blot results confirmed the positive ELISA findings, and immune serum antibodies recognized several viral proteins. The neutralization assay (PRNT) showed visible viral neutralization, which was in accordance with the ELISA and Western Blot results.

**Conclusions.:**

The findings suggest that hyperimmune llama serum could constitute a source of therapeutic antibodies against SARS-CoV-2 infections (lineage B.1.1), and should be studied in further research.

## INTRODUCTION

SARS-CoV-2, which causes coronavirus disease (COVID-19), emerged in late 2019 in Wuhan, China, triggering a pandemic with more than 765 million confirmed cases and more than 6.9 million deaths ^1^. This virus, similar to the coronavirus responsible for the severe acute respiratory syndrome (SARS-CoV) and the Middle East respiratory syndrome (MERS-CoV), can produce severe respiratory illness characterized by fever, shortness of breath, and lung infiltration and inflammation ^2^^,^^3^. However, unlike its predecessors, SARS-CoV-2 spreads efficiently, causing asymptomatic cases and favoring its global dissemination; therefore, it is expected to remain for a long time or to become an endemic disease. One of the important reasons to consider that COVID-19 will become an endemic disease in the future is that it has been demonstrated that SARS-CoV-2 is capable of accumulating genetic mutations, generating new genetic variants of the virus (Alpha, Beta, Gamma, Delta and Omicron) that are capable of evading prevention by existing vaccines or treatments ^1^^,^^4^.

Currently, there are few drugs approved for the clinical treatment of this disease, and some of them are still in clinical trials ^4^^,^^5^. The pandemic has raised the urgent need for effective, specific and rapidly accessible drugs against COVID-19 ^6^^,^^7^. Previous studies reported the potential usefulness of human plasma from convalescent individuals to treat COVID-19 ^8^^-^^10^; however, plasma from convalescent individuals is difficult to find and time-consuming to produce because of the need to screen convalescent individuals and a costly process to ensure a highly efficient, high-quality product approved for human use. Currently, no diagnostic protocol is available to detect hyperimmune individuals on a routine basis, and generating large quantities of this product to meet clinical demand is unfeasible ^10^.

It is important to consider the use of some large animals (horse, sheep or llamas) as a research source of therapeutic antibodies in order to explore treatment alternatives for COVID-19 other than human plasma. The animal approach is still widely used today in the treatment of snake, scorpion and spider sting envenomation ^11^^,^^12^; this process consists of collecting antibody-rich serum after injecting animal toxins into horses; and although this method may seem old-fashioned, it is still used worldwide, and it saves lives ^12^.

Although animal-derived immunoglobulins provide effective treatments in some cases such as snakebite envenomation, we must also consider the risk associated with this method; as intravenous administration of animal-derived immunoglobulins is associated with the development of early and late adverse reactions and anaphylaxis, known as serum sickness. In the context of animal-derived antibodies with neutralizing activity against pathogens and/or toxins, a recent study reported that antisera from horses can effectively neutralize SARS-CoV-2 *in vitro*^13^.

Some camelid species such as the llama (*Lama glama*) are of special interest in the study of venom neutralizing sera because of its great capacity to adapt to many climatic environments and for being a docile animal. In a previous study, we demonstrated the usefulness of immunization-induced hyperimmune llama serum to neutralize the toxic effects and lethality of the venom of *Bothrops atrox* in mice ^14^. Although the use of animals in medicine has declined in recent years, the scale of the current crisis requires a multi-pronged approach that inevitably involves the holistic and complementary use of animals to control SARS-CoV-2 and future emerging viruses that affect both humans and the animals with which they coexist. Therefore, the aim of this study was to evaluate the serological antibody response of a llama (*Lama glama*) to immunization with SARS-CoV-2 (lineage B.1.1) and the neutralizing capacity of hyperimmune llama serum against SARS-CoV-2 (lineage B.1.1) in Vero cells.

**KEY MESSAGES**

Motivation for the study. Currently, there are few studies on the use of animals as potential sources of SARS-CoV-2 neutralizing sera or antibodies; therefore, research in this area is needed to address the problem.

Major findings. Hyperimmune serum from llama (*Lama glama*) is capable of neutralizing SARS-CoV-2 virus (B.1.1 lineage) infection in Vero cell culture.

Implications. The results of this study suggest that immune llama serum could be a potential source of SARS-CoV-2 (lineage B.1.1)-neutralizing antibodies for use in further preclinical and clinical research on the treatment of COVID-19.

## MATERIALS AND METHODS

### Study design

We carried out an experimental study. A llama was immunized with inactivated SARS-CoV-2 (Lineage B.1.1) and the antibody response was evaluated by ELISA and Western Blot of the immune serum; viral neutralization in cell culture was evaluated by the Plate Reduction Neutralization Test (PRNT).

### Biological material

*Experimental animal*

A three-year-old male llama (Lama glama) specimen was fed with alfalfa hay, concentrate and water ad libitum. Prior to experimentation, the animal underwent a 40-day veterinary evaluation and quarantine period. The animal was obtained from agricultural center (SAIS Pachacutec); it was healthy and under continuous veterinary follow-up during the experimental period.

*SARS-CoV-2 virus*

The SARS-CoV-2 strain (lineage B.1.1) was provided by the viral isolation area of the National Referral Laboratory for Respiratory Viruses of the National Institute of Health (INS), Peru.

*Vero cells*

We used the cell line Vero 81 ATCC CCL-81 TM, derived from the kidney of the African green monkey (*Cercopithecus aethiops*), used by several research institutions for the isolation of SARS-CoV-2. The Vero 81 cell line came from the cell bank of the National Public Health Center of the INS, Peru.

### Procedures

*SARS-CoV-2 collection*

**Figure 1:**
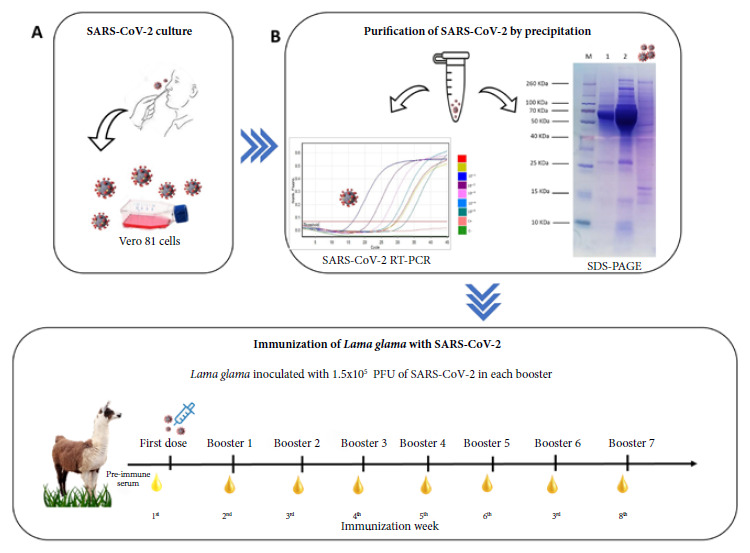
A) Isolation of SARS-CoV-2. (B) Purification of SARS-CoV-2 : Precipitation-purified virus was analyzed by real-time PCR and SDS-PAGE (M) Protein molecular weight marker (Thermo Scientific #26623). (1) Culture supernatant of SARS-CoV-2 virus. (2) Culture maintenance medium. (3) Precipitation-purified SARS-CoV-2 virus (2.5 M NaCl + 20% PEG 6000). (C) Scheme of llama immunizations. The llama was immunized with purified heat-inactivated SARS-CoV-2 virus. A first dose and 7 boosters were applied.

SARS-CoV-2 was obtained from a previously filtered reverse transcriptase polymerase chain reaction (RT-PCR)-positive nasopharyngeal swab sample, which was then inoculated into Vero 81 cells (Figure 1A). The virus was isolated in a Biosafety Level 3 Laboratory (BSL-3) following established protocols; supernatants were centrifuged, aliquoted and quantified by plaque titration method counting the Plate Forming Units (PFU).

The culture supernatant was precipitated with 20% PEG + 2.5 M NaCl, centrifuged, and the pellet was resuspended in 1 mL of the final volume of 1X HEPES buffer pH 7.4. Viral RNA was extracted using the Maxwell 16 Viral Total Nucleic Acid Purification Kit (Promega), and amplified with the AllplexTM SARS CoV2 Assay kit from Seegene. The virus was also characterized by genomic sequencing to determine its genotype or genetic lineage. 

The protein profile of viral culture supernatants and purified virus were analyzed on 15% polyacrylamide gels using the Spectra™ Multicolor protein marker (Thermo Scientific # 26623).

*Immunization of Lama glama with SARS-CoV-2*

Immunizations of the llama were carried out with previously purified and heat-inactivated SARS-CoV-2 as inoculum. A dose of 1.5 x 10^5^ PFU/mL of virus was applied as inoculum, in a 1:1 ratio mixture between virus and adjuvant (FAMA # 3030-GERBU). The total volume of inoculum, 2 mL (1 mL of virus and 1 mL of adjuvant) was injected subcutaneously into the dorsum of the llama, distributing the inoculum proportionally to four locations on the animal’s dorsum, and alternating between both sides of the animal’s back between each inoculation. The immunization schedule consisted of a first dose and seven boosters with the same dose of purified and inactivated SARS-CoV-2 at a weekly interval (Figure 1).

*ELISA for the measurement of Lama glama antibodies against SARS-CoV-2 virions.*

Ten mL of whole blood samples were obtained. Each sample was extracted before the first and after each of the following immunizations, in order to collect pre-immune and immune serum. The complete SARS-CoV-2 viral antigen was diluted in carbonate buffer (0.015 M Na_2_CO_3_, 0.035 M NaHCO_3_) to a final concentration of 3 x 10^5^ PFU/mL. Then, 100 µL/well of diluted viral antigen were placed in a plate and incubated overnight at 4°C. Excess antigen was removed, 250 µL of blocking buffer (PBS 1X with 2% skim milk) was added, and then it was incubated at room temperature (RT) for 1 hour. The sera to be titered were diluted from 1:200 to 1:1600 in serum and conjugate dilution buffer (TSC: 1X PBS with 0.1% skim milk and 0.05% Tween 20), as well as negative control (pre-immune serum from the llama before immunizations) and blank (carbonate buffer without antigen). Excess blocking buffer was removed and washed five times with wash buffer (TL: 1X PBS, 0.05% Tween 20), then 100 µL of the diluted serum was added to each well, the sera were run in duplicate, and incubated for one hour at RT.

Excess serum was removed and washed five times with TL and then 100 µL of conjugate (llama anti-IgG antibody labeled with (H+L)-Peroxidase) diluted 1: 5000 in TSC were added; then it was incubated for one hour at RT, removed the excess conjugate and washed five times with TL, 100 µL of 3,3’,5,5’-tetramethylbenzidine (TMB) substrate were added to each well, and then incubated for 15 minutes at RT in the dark. Finally, 50 µL of stop solution (0.5 M H_2_SO_4_) was added to each well. The plate was read in a spectrophotometer at a wavelength of 450 nm.

*Western Blot*

The complete SARS-CoV-2 lysate was analyzed by sodium dodecylsulfate polyacrylamide gel electrophoresis (SDS-PAGE) on a 12% polyacrylamide gel with 1X tris glycine buffer; then the gel was transferred to a polyvinylidene difluoride (PVDF) membrane (Thermo Scientific # 88518) previously activated with absolute methanol. The transfer process was carried out at 25 V for 40 min using transfer buffer (25 mM tris, 192 mM glycine, 20% methanol, 0.05% SDS) in a Trans-Blot SD semi-dry transfer cell (Biorad). The membrane was placed in blocking solution (1X PBS, 0.1% Tween 20) and incubated for one hour at 60 rpm. Then, the membrane was placed in a 1/1000 dilution of SARS-CoV-2 immunized hyperimmune llama serum diluted in blocking buffer for one hour at 60 rpm. The membrane was removed from the serum dilution and placed in wash solution (1X PBS, Tween 20) for 5 minutes at 60 rpm; this washing process was performed three times. The membrane was placed in a 1/5000 dilution of peroxidase-conjugated llama anti-IgG antibody diluted in blocking buffer for one hour at 60 rpm. The membrane was placed on a clear plastic base, then detection solution (Western blot ultrasensitive HRP substrate-TAKARA), prepared according to the manufacturer’s recommendations, was added; the chemiluminescent signal was detected using the Chemidoc XRS+ detection system (BioRad).

*Plaque reduction viral neutralization test*

Neutralizing antibody levels are measured by PRNT, which is considered the gold standard test for measuring neutralizing antibodies in a serum ^15^. Vero 81 cells (1.8 × 10^5^ cells per well) were seeded in 24-well cell culture plates in EMEM medium supplemented with 10% FBS and incubated for 24 hours at 37°C. Llama serum samples were inactivated by heat at 56°C for 30 minutes and serial dilutions were incubated with SARS-CoV-2 stock (40-50 PFU) for 1 hour at 37°C. The virus-serum mixture and controls (control virus, dilution medium) were inoculated into Vero 81 cells and incubated at 37°C for 1 hour 30 minutes, and then 1 mL of 0.75% carboxymethylcellulose overlay medium was added. Cells were incubated at 37 °C in 5% CO_2_ for five days. Cells were stained with crystal violet and the PFU were quantified for PRNT50 titer calculation. Each serum sample was analyzed in duplicate.

### Statistical analysis

We used R Studio statistical software to calculate the PRNT50 titer by probit analysis with 95% confidence interval (CI) ^15^. Microsoft Excel 2016 was used for the comparison analysis between ELISA and PRNT assays.

### Ethical Aspects

The study was approved by the Institutional Ethics Committee for the Use of Experimental Animals of the National Institute of Health of Peru (Code OI-040-20).

## RESULTS

The SARS-CoV-2 culture had a viral titer of 1.5 x 10^6^ PFU/mL. Purification of the virus by precipitation resulted in an opaque suspension after resuspending the precipitate. Virions were inactivated by heating the sample at 56°C for one hour, and verified by culture (data not shown); and this purification was verified by analyzing the virions by RT-PCR; noting the detection of specific RdRP and S genes confirming the identity of SARS-CoV-2 virus (Figure 1B). Genomic sequencing determined that the genetic lineage of the cultured virus was the B.1.1 lineage.

The SDS-PAGE electrophoresis analysis showed that the culture supernatant of SARS-CoV-2 culture, as well as the culture medium alone, contained abundant proteins besides virion proteins. After the virions were purified by precipitation, the other proteins in the culture medium were removed and only SARS-CoV-2 proteins remained (Figure 1B).

**Figure 2:**
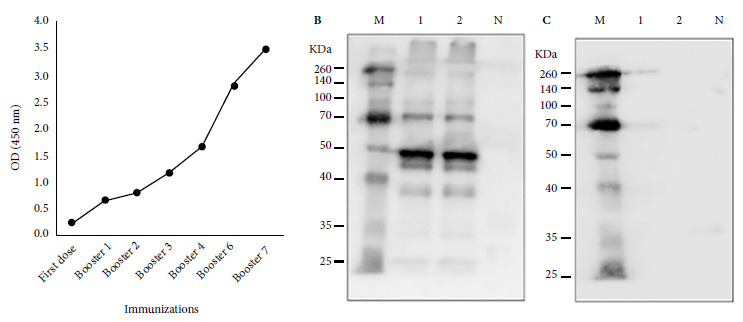
Llama antibody response to SARS-CoV-2 immunization. Antibodies against SARS-CoV-2 virus were measured by ELISA assay in llama serum after booster (A). Purified and inactivated SARS-CoV-2 virions were used as antigen for ELISA. Western blot analysis (B and C) used whole virus protein lysate (lanes 1 and 2), anti-llama IgG conjugate with peroxidase and BSA as negative protein (lane N). (B) Hyperimmune llama serum as primary antibody. (C) SARS- CoV-2 negative human serum as primary antibody. (M) Protein molecular weight marker (Thermo Scientific # 26623).

**Figure 3:**
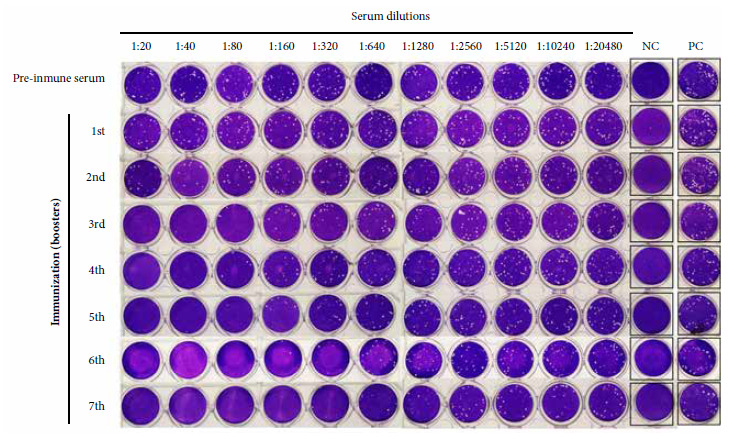
Plaque Reduction Neutralization Test (PRNT) of hyperimmune llama serum. NC: Negative control (cells only, no virus), PC: Virus control.

**Figure 4:**
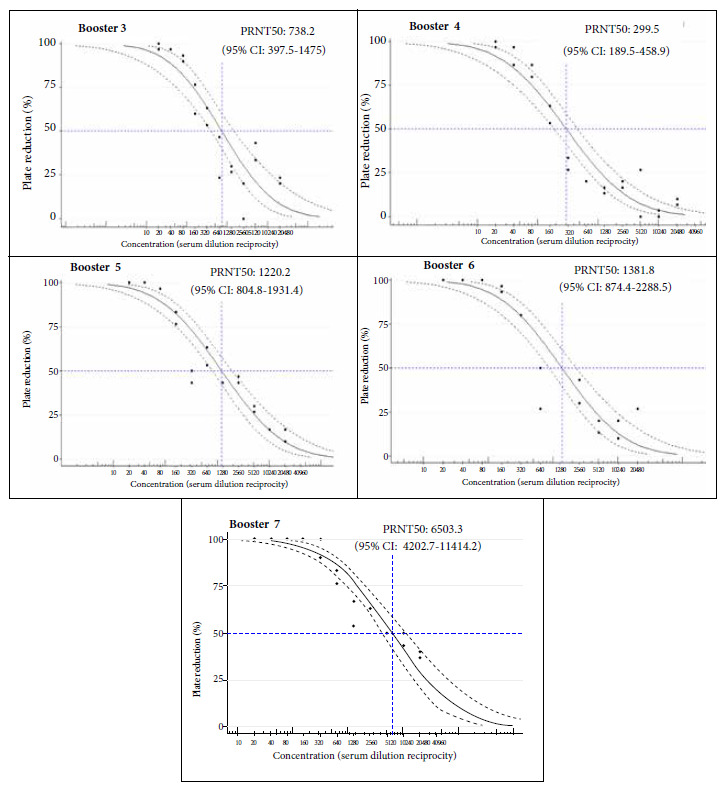
Analysis of the results of the Plaque Reduction Neutralization Test (PRNT). The PRNT50 value was calculated from the plaque reduction counts and is indicated after each immunization booster (Boosters 3 to 7).

**Figure 5:**
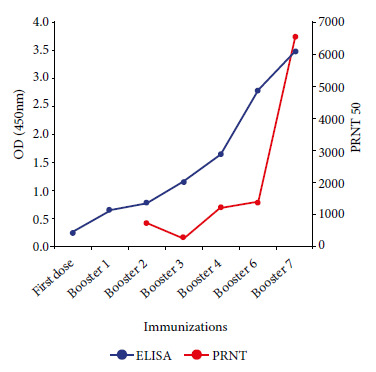
Comparison analysis between ELISA and PRNT assays.

The ELISA to detect antibodies against SARS-CoV-2 in llama serum revealed that IgG antibody titers increased in the serum samples in accordance with increasing immunization boosters; this increase in antibody response was most evident from the fourth week after the start of immunizations, reaching a maximum after the seventh booster was applied (Figure 2A). Western Blot results showed reactivity of the total SARS-CoV-2 antigen to immune llama serum obtained after the seventh booster immunization with SARS-CoV-2; furthermore, no reaction of the virus antigens with serum from a healthy person (negative control) was observed (Figure 2B). The response to immunization, assessed by ELISA and PRNT assays, showed a progressive increase in antibodies to SARS-CoV-2 as immunizations increased (Figures 2-5). We found an increase in virus neutralization by the hyperimmune serum obtained after the highest number of immunizations, evidenced by the absence or reduction of viral plaque counts in the PRNT neutralization assay for sera collected after the fourth week of immunization (Figure 3).

The potency or viral neutralizing capacity of the llama serum is measured by the PRNT50 values of the PRNT assay, and these values were higher as the immunization doses increased, which correlates with the antibody titers of the studied sera (Figure 4-5). The maximum PRNT50 value achieved after the seven boosters was PRNT50 = 6506.3 (95%CI 4202.7-11414.2).

## DISCUSSION

The present study analyzed the immune response of llama to immunization with SARS-CoV-2 virus (B.1.1 lineage), and the ability of this hyperimmune serum to neutralize the virus infection (B.1.1 lineage) in a Vero cell culture; showing encouraging results in the investigation of new possible treatments of COVID-19 based on camelid nanoantibodies. Our results show that there was a significant increase in seroreactivity to SARS-CoV-2 (B.1.1 lineage) after the fourth week, with higher antibody titers after the seventh immunization boost or eighth week. Also, the PRNT assay showed visible viral neutralization, which was concordant with the ELISA and Western Blot results. This suggests that hyperimmune llama serum could be used as a possible source of therapeutic antibodies against SARS-CoV-2 infections.

Currently, there is no clearly defined treatment for COVID-19, although there are studies on antibodies capable of neutralizing or inhibiting virus infection ^16^^,^^17^. At the moment, the most effective control strategy worldwide is vaccination. However, the continuous emergence of new genetic variants represents a challenge to both treatments and existing vaccines ^1^^,^^4^.

Some camelid species such as the llama (*Lama glama*) and the camel (*Camelus* sp.) are of special interest in the study and production of venom neutralizing sera ^18^^,^^19^. Previous studies have described the ability of immune llama serum to neutralize the venoms of some snake species ^14^, and of camelid serum to neutralize scorpion venoms ^18^^,^^19^.

The different studies on SARS-CoV-2 require having the virus in pure form, either in its active form for infection studies, or the inactivated virus for use as antigen, for example in immunizations ^20^^,^^21^; for this purpose, the cellular debris and the medium component must be removed from the viral cultures, so that they do not interfere with the experimental procedures. Most protocols for virus concentration and purification use a process of virions precipitation from the culture using saturated solutions; in our study, a saturated solution of polyethylene glycol and sodium chloride was used to precipitate virions from the supernatant of the SARS-COV-2 culture in Vero cells, and this precipitate was resuspended in HEPES buffer.

Purified virions should also be inactivated for experimental procedures that do not involve virus infection or do not require the virus in its active form, to avoid the risk of infection of personnel. Different inactivation methods have been documented such as inactivation by gamma radiation, UV-C light, formaldehyde, and glutaraldehyde ^20^^-^^23^. In this study we used two methods of virus inactivation, beta-propiolactone and heat inactivation, both methods were effective, but heat inactivation was chosen to avoid the risk of chemically modifying the virions, or toxicity by beta-propiolactone; since these virions would be used for llama immunizations.

Freund’s adjuvant is the most commonly used adjuvant for immunization in many studies. We used GERBU FAMA adjuvant ^14^, which has been designed for immunization of older animals to produce a better immune response and fewer allergic reactions or adverse effects. In addition, subcutaneous immunization of each dose was used at different inoculation sites, since it has been reported to produce a better immune response. The GERBU FAMA adjuvant produced a good immune response as shown by ELISA and Western Blot results.

The immune response of the llama after immunizations with the virus was evidenced by increased titers of antibodies specific for SARS-CoV-2, starting from the fourth week of immunization, measured by ELISA and PRNT assays, using inactivated SARS-CoV-2 virions as antigen. Western blot analysis recognized SARS-CoV-2 total antigen proteins by the total antibodies to the virus present in serum obtained after the seventh immunization boost. Since the immunizations were performed with purified and inactivated whole SARS-CoV-2 virus, the immune response of the llama produced antibodies against different proteins of the virus, particularly for the most immunogenic or abundant proteins; for this reason, the Western Blot assay recognized several viral proteins by the antibodies present in the hyperimmune serum.

Our results show the neutralization of the B.1.1 SARS-CoV-2 lineage by sera collected after the fourth week of immunization; with progressively increasing PRNT50 neutralization values at higher immunization doses, PRNT50 = 1:6506.3 (95% CI: 4202.7-11414.2). Our hyperimmune llama serum could also serve as a source for the purification of specific antibodies directed to key viral proteins such as Spike or RBD; or even to isolate monoclonal antibodies from the immunized llama.

Different animal species have been immunized in different studies aimed at producing specific antibodies, such as shrews, transgenic mice expressing human ACE2, hamsters ^24^^,^^25^^)^ and alpacas ^26^^,^^27^; but these immunizations are generally performed with pure antigens or recombinant proteins such as the spike protein. In our study, similar to other studies on camelids with SARS-CoV-2 ^26^^,^^27^, immunization of a llama with full SARS-CoV-2 virus has been shown to generate antibodies that can recognize SARS-CoV-2 antigens (B.1.1 lineage) and neutralize infection in Vero cells.

Initially, since there were no treatments for COVID-19, the use of immune plasma from people who had overcome COVID-19 infection was proposed as a possible therapy, obtaining positive and negative results in preliminary studies ^8^^-^^10^. Subsequently, monoclonal antibodies have been isolated from the blood of infected patients that show neutralizing activity against SARS-CoV-2 infection ^28^^,^^29^; some of these, such as REGN-COV2, a mAb cocktail, have been approved by the US Food and Drug Administration (FDA) for the treatment of COVID-19 ^30^. But some of these monoclonal antibody-based drugs have also been losing efficacy due to the emergence of genetic variants of SARS-CoV-2 virus (Alpha, Beta, Gamma, Delta and Omicron). Currently, there are already FDA-approved drugs aimed at treating vulnerable persons with mild or moderate cases of COVID-19 (remdesivir, paxlovid and lagevrio, among others) ^30^.

Complete hyperimmune llama serum contains a large number of antibodies, only a fraction of which are neutralizing antibodies; therefore, the utility of immune serum with SARS-CoV-2 neutralizing activity lies in the potential to identify and isolate antibodies that neutralize specific virus targets, such as the spike protein and its RBD domain, which is the protein that binds to the ACE2 cell receptor and enables SARS-CoV-2 infection. In our study, hyperimmune llama serum reacted to whole SARS-CoV-2 protein bands in Western Blot analysis; suggesting that immunizations with the whole virus also induced antibodies against the spike protein in serum; this could partly explain the ability of immune serum to neutralize SARS-CoV-2 in Vero cell culture, as the spike protein is key to virus infection in cells.

One of the limitations of this study is that we worked with total antibodies and were not able to purify or isolate SARS-CoV-2-specific neutralizing antibodies. In addition, more SARS-CoV-2 variants were not included in this study due to the complexity of culturing them in a type 3 biosafety environment and the difficulty of standardizing neutralization tests in cell culture for each variant of the virus. Finally, we only identified SARS-CoV-2 (lineage B.1.1), therefore, future studies are required to demonstrate whether the same effect could be observed in other lineages or variants.

In conclusion, our study demonstrated that immunization of a llama (*Lama glama*) with SARS-CoV-2 virus (lineage B.1.1) elicits a specific antibody immune response, and that antibodies in hyperimmune serum have viral neutralizing activity in Vero cell culture. Our findings reveal that llamas could be useful animals for the therapeutic evaluation of antibodies against SARS-CoV-2 virus infection (lineage B.1.1).
