# Supplementary material for: Neutralization of SARS-CoV-2 (lineage B.1.1) by hyperimmune llama (Lama glama) serum in vero cell culture
Source: Rev Peru Med Exp Salud Publica. 2023 Sep 27;40(3):287–96. doi: 10.17843/rpmesp.2023.403.12509 (PMC10953648; doi:10.17843/rpmesp.2023.403.12509)
Supplement: Translation

# Translation: Neutralización del virus SARS-CoV-2 (linaje B.1.1) por suero hiperinmune de llama (*Lama glama*) en cultivo de células Vero

**Authors:** Verónica Yaniro, Silvia Capristano, Henri Bailon, Juan Lévano, Marco Galarza, David García, Omar Cáceres, Carlos Padilla, Harrison Montejo, Paquita García, Mary Celis, Silvia Seraylan, Yessica Garayar, Miryam Palomino

**Affiliations:** 1 Laboratorio de Referencia Nacional de Biotecnología y Biología Molecular, Centro Nacional de Salud Pública, Instituto Nacional de Salud, Lima, Perú.; 2 Laboratorio de Referencia Nacional de Infecciones Metaxénicas Virales, Centro Nacional de Salud Pública, Instituto Nacional de Salud, Lima, Perú.; 3 Laboratorio de Referencia Nacional de Virus Respiratorios, Centro Nacional de Salud Pública, Instituto Nacional de Salud, Lima, Perú.; 4 Centro Nacional de Producción de Biológicos, Instituto Nacional de Salud, Lima, Perú.

**Keywords:** Coronavirus, SARS-CoV-2, Lama glama, Pruebas de Neutralización, Sueroterapia para COVID-19

## Abstract

**Objetivo.:**

Evaluar la respuesta serológica de anticuerpos de una llama (*Lama glama*) a la inmunización del virus SARS-CoV-2 (linaje B.1.1) y la capacidad neutralizante del suero de llama hiperinmune frente al virus SARS-CoV-2 (linaje B.1.1) en células Vero.

**Materiales y métodos.:**

Se inmunizó una llama con el virus SARS-CoV-2 inactivado (Linaje B.1.1) y se analizaron muestras de suero para evaluar el nivel de anticuerpos mediante ELISA, así como la reactividad a antígenos de SARS-CoV-2 mediante Western Blot. Además, se evaluó la neutralización viral en cultivos celulares por la Prueba de Neutralización por Reducción de Placas (PRNT, por sus siglas en inglés).

**Resultados.:**

Se observó un aumento en la serorreactividad en la llama inmunizada desde la semana 4 en adelante. Los títulos de anticuerpos fueron más elevados en el séptimo refuerzo de inmunización. Los resultados de Western Blot confirmaron los hallazgos positivos del ELISA, y los anticuerpos del suero inmune reconocieron varias proteínas virales. El ensayo de neutralización (PRNT) mostró una neutralización viral visible, concordante con los resultados de ELISA y Western Blot.

**Conclusiones.:**

Los hallazgos sugieren que el suero hiperinmune de llama podría constituir una fuente de anticuerpos terapéuticos contra las infecciones por el virus SARS-CoV-2 (linaje B.1.1) y que deberá ser evaluado en estudios posteriores.

## INTRODUCCIÓN

El virus SARS-CoV-2, que causa la enfermedad por coronavirus (COVID-19), surgió a finales de 2019 en Wuhan, China, desencadenando una pandemia con más de 765 millones de casos confirmados y más de 6,9 millones de fallecimientos ^1^. Este virus, similar al coronavirus responsable del síndrome respiratorio agudo severo (SARS-CoV) y del síndrome respiratorio de Oriente Medio (MERS-CoV), puede producir una enfermedad respiratoria grave caracterizada por fiebre, dificultad para respirar e infiltración pulmonar e inflamación ^2^^,^^3^. Sin embargo, a diferencia de sus predecesores, el virus SARS-CoV-2 se propaga eficazmente, provocando casos asintomáticos y favoreciendo su diseminación mundial; por lo que, se espera permanezca por mucho tiempo o se convierta en una enfermedad endémica. Una de las razones importantes para considerar que la COVID-19 se convertirá en una enfermedad endémica en el futuro es que ya se ha demostrado que el virus SARS-CoV-2 es capaz de acumular mutaciones genéticas, generando nuevas variantes genéticas del virus (Alfa, Beta, Gama, Delta y Ómicron) que son capaces de evadir la prevención por las vacunas o tratamientos existentes ^1^^,^^4^.

En la actualidad, existen escasos fármacos aprobados para el tratamiento clínico de esta enfermedad, y algunos de ellos, aún se encuentran en ensayos clínicos ^4^^,^^5^. Esta pandemia ha planteado la necesidad urgente de medicamentos efectivos, específicos y rápidamente accesibles contra la COVID-19 ^6^^,^^7^. Estudios previos informaron la utilidad potencial del plasma humano de personas convalecientes para tratar la COVID-19 ^8^^-^^10^; sin embargo, el plasma de personas convalecientes es difícil de encontrar y requiere mucho tiempo producirlo debido a la necesidad de detectar personas convalecientes y un proceso costoso para garantizar un producto con alta eficiencia y calidad, aprobado para uso humano. Actualmente, no se dispone de un protocolo de diagnóstico para detectar individuos hiperinmunes de forma rutinaria, y generar grandes cantidades de este producto para satisfacer la demanda clínica resulta inviable ^10^.

Para explorar alternativas de tratamiento para la COVID-19 distintas al plasma humano, es importante considerar el uso de algunos animales mayores (caballo, oveja o llamas) como fuente de investigación de anticuerpos terapéuticos; pues el enfoque animal todavía se usa ampliamente hoy en día en el tratamiento del envenenamiento por picaduras de serpientes, escorpiones y arañas ^11^^,^^12^; a través de un proceso que consiste en recolectar suero rico en anticuerpos después de inyectar las toxinas animales en los caballos; y aunque este método puede parecer anticuado, todavía se usa en todo el mundo y salva vidas ^12^.

Aunque las inmunoglobulinas de origen animal brindan tratamientos efectivos para algunos casos como el envenenamiento por picaduras de serpientes, también debemos considerar el riesgo que conlleva este método; pues la administración intravenosa de inmunoglobulinas derivadas de animales está asociada con el desarrollo de reacciones adversas tempranas y tardías y anafilaxia, conocida como enfermedad del suero. En este contexto de anticuerpos derivados de animales con actividad neutralizante contra patógenos y/o toxinas; un estudio reciente informó que los antisueros generados en caballos pueden neutralizar eficazmente el virus SARS-CoV-2 *in vitro*^13^.

Algunas especies de camélidos como la llama (*Lama glama*) son de especial interés en el estudio de sueros neutralizantes de venenos; además, es utilizada debido a su gran capacidad de adaptación a muchos ambientes climáticos y por ser un animal dócil. En un estudio previo, demostramos la utilidad del suero de llama hiperinmune inducido por inmunización, para neutralizar los efectos tóxicos y la letalidad del veneno de la serpiente *Bothrops atrox* en ratón ^14^. Aunque el uso de animales en medicina ha disminuido en los últimos años, la escala de la crisis actual requiere un enfoque múltiple que inevitablemente implica el uso holístico y complementario de animales para controlar al virus SARS-CoV-2 y los futuros virus emergentes que afectan tanto a los humanos como los animales con los que conviven. Por lo tanto, el objetivo de este estudio fue evaluar la respuesta serológica de anticuerpos de una llama (*Lama glama*) a la inmunización con el virus SARS-CoV-2 (linaje B.1.1) y la capacidad neutralizante del suero de llama hiperinmune frente al virus SARS-CoV-2 (linaje B.1.1) en células Vero.

**MENSAJE CLAVE**

Motivación para realizar el estudio. Actualmente, existen pocos estudios sobre el uso de animales como potenciales fuentes de sueros o anticuerpos neutralizantes del virus SARS-CoV-2; por ello es necesario realizar investigaciones en esta área para abordar el problema.

Principales hallazgos. El suero hiperinmune de la llama (*Lama glama*) es capaz de neutralizar la infección del virus SARS-CoV-2 (linaje B.1.1) en cultivo de células Vero.

Implicancias. Los resultados de este estudio sugieren que el suero inmune de llama podría ser una fuente potencial de anticuerpos neutralizantes del virus SARS-CoV-2 (linaje B.1.1), para ser usados en otras investigaciones preclínicas y clínicas sobre el tratamiento de la COVID-19.

## MATERIALES Y MÉTODOS

### Diseño de estudio

El presente estudio fue de tipo experimental. Se inmunizó una llama con el virus SARS-CoV-2 inactivado (Linaje B.1.1) y se evaluó la respuesta de anticuerpos por ensayo de ELISA y Western Blot en el suero inmune; y se evaluó la neutralización viral en cultivo celular por la Prueba de Neutralización por Reducción de Placas (PRNT, por sus siglas en inglés).

### Material biológico

*Animal de experimentación*

Se utilizó un espécimen de llama (*Lama glama*) macho de tres años que fue alimentado con heno de alfalfa, concentrado y agua *ad libitum*. Previo a la experimentación pasó un periodo de evaluación veterinaria y cuarentena de 40 días. El animal procedía de un centro agropecuario (SAIS Pachacútec), se encontraba sano y estuvo en seguimiento veterinario continuo durante el período de experimentación.

*Virus SARS-CoV-2*

La cepa del virus SARS-CoV-2 (linaje B.1.1) fue proporcionada por el área de aislamiento viral del Laboratorio Nacional de Referencia de Virus Respiratorios del Instituto Nacional de Salud (INS), Perú.

*Células Vero*

Utilizamos la línea celular Vero 81 ATCC CCL-81 ^TM^, derivada del riñón del mono verde africano (*Cercopithecus aethiops*), utilizada por diversas instituciones de investigación en el aislamiento del virus SARS-CoV-2. La línea celular Vero 81 provino del Banco de células del Centro Nacional de Salud Pública del INS, Perú.

### Procedimientos

*Obtención del virus SARS-CoV-2*

El virus SARS-CoV-2 obtenido a partir de una muestra de hisopado nasofaríngeo, con resultado positivo por reacción en cadena de la polimerasa con transcriptasa inversa (RT-PCR, por sus siglas en inglés), previamente filtrada, se inoculó en células Vero 81 (Figura 1A). El virus fue aislado en un Laboratorio de Bioseguridad Nivel 3 (BSL-3, por sus siglas en inglés), siguiendo los protocolos establecidos; los sobrenadantes se centrifugaron, se alicuotaron y se cuantificó por el método de titulación de placa contando las Unidades Formadoras de Placa (UFP).

**Figura 1:** Purificación del virus SARS-CoV-2 e inmunización de llama. (A) Aislamiento del virus SARS-CoV-2. (B) Purificación del virus SARSCoV-2: El virus purificado por precipitación se analizó por PCR en tiempo real y SDS-PAGE (M) Marcador de peso molecular de proteínas (Thermo Scientific #26623). (1) Sobrenadante de cultivo del virus SARS-CoV-2. (2) Medio de mantenimiento del cultivo. (3) Virus SARS-CoV-2 purificado por precipitación (2,5 M NaCl + 20% PEG 6000). (C) Esquema de inmunizaciones de la llama. La llama fue inmunizada con el virus SARS-CoV-2 purificado e inactivado por calor. Se le aplicó una primera dosis y 7 refuerzos.

El sobrenadante del cultivo se precipitó con PEG 20% + NaCl 2,5 M, se centrifugó, y el sedimento se resuspendió en 1 mL del volumen final de tampón HEPES 1X pH 7,4. Se extrajo el ARN viral, usando el Maxwell 16 Viral Total Nucleic Acid Purification Kit (Promega), y fue amplificado usando el kit Allplex^TM^ SARS CoV2 Assay de Seegene. Este virus también fue caracterizado por secuenciamiento genómico para determinar su genotipo o linaje genético.

El perfil de proteínas de los sobrenadantes de cultivos virales y el virus purificado se analizaron en geles de poliacrilamida al 15%, usando el marcador de proteínas Spectra™ Multicolor (Thermo Scientific # 26623).

*Inmunización de Lama glama con virus SARS-CoV-2*

Las inmunizaciones de la llama se realizaron con el virus SARS-CoV-2 previamente purificado e inactivado por calor como inóculo. Se aplicó como inóculo una dosis de 1,5 x 10^5^ UFP/mL del virus, en una mezcla de proporción 1:1 entre el virus y el adyuvante (FAMA # 3030-GERBU). El volumen total del inóculo, 2 mL (1 mL de virus y 1 mL de adyuvante) se inyectó por vía subcutánea en el dorso de la llama, distribuyendo el inóculo proporcionalmente en cuatro lugares del dorso del animal, y alternando entre ambos lados del lomo del animal entre cada inoculación. El calendario de inmunización consistió en una primera dosis y siete refuerzos con la misma dosis del virus SARS-CoV-2 purificado e inactivado en un intervalo semanal (Figura 1).

*ELISA para la medición de anticuerpos de Lama glama contra viriones SARS-CoV-2*

Se obtuvieron 10 mL de muestras de sangre total, antes de la primera y después de cada una de las inmunizaciones, para la colecta de suero preinmune e inmune. El antígeno viral SARS-CoV-2 completo se diluyó en tampón de carbonato (0,015 M Na_2_CO_3_, 0,035 M NaHCO_3_) hasta una concentración final de 3 x 10^5^ UFP/mL. A la placa se le aplicó 100 µL/pocillo del antígeno viral diluido, y se incubó toda la noche a 4° C. Se retiró el exceso de antígeno, se añadieron 250 µL de tampón de bloqueo (PBS 1X con 2% leche descremada), se incubó a temperatura ambiente (TA) durante 1 hora. Los sueros a titular se diluyeron de 1:200 a 1:1600 en el tampón de dilución de suero y conjugado (TSC: PBS 1X con leche descremada 0,1% y Tween 20 0,05%), así como el control negativo (suero pre-inmune de la llama antes de las inmunizaciones) y blanco (tampón de carbonato sin antígeno). Se retiró el tampón de bloqueo excedente y se lavó cinco veces con tampón de lavado (TL: 1X PBS, Tween 20 al 0,05%), luego se agregó 100 µL del suero diluido a cada pocillo, los sueros se corrieron por duplicado, y se incubó durante una hora a TA. Se retiró el suero excedente y se lavó cinco veces con TL y luego se agregó 100 µL de conjugado (anticuerpo anti-IgG de llama marcado con (H+L)-Peroxidasa) diluido 1:5000 en TSC; y se incubó durante una hora a TA, se retiró el exceso del conjugado y se lavó cinco veces con TL, se añadió 100 µL de sustrato 3,3′,5,5′-tetrametilbencidina (TMB) a cada pocillo, y se incubó durante 15 minutos a TA en oscuridad. Finalmente, se añadió a cada pocillo 50 µL de solución de parada (0,5 M de H_2_SO_4_). La placa se leyó en un espectrofotómetro a una longitud de onda de 450 nm.

*Western Blot*

El lisado del virus SARS-CoV-2 completo se analizó mediante electroforesis en gel de poliacrilamida con dodecilsulfato sódico (SDS-PAGE, por sus siglas en inglés) en un gel de poliacrilamida al 12%, con tampón tris glicina 1X; y el gel se transfirió a una membrana de difluoruro de polivinilideno (PVDF) (Thermo Scientific # 88518) previamente activada con metanol absoluto. El proceso de transferencia se realizó a 25 V durante 40 minutos usando tampón de transferencia (tris 25 mM, glicina 192 mM, metanol al 20%, SDS al 0,05%) en una celda de transferencia semiseca Trans-Blot SD (Biorad). La membrana se colocó en solución de bloqueo (PBS 1X, Tween 20 0,1%) y se incubó durante una hora a 60 rpm. Luego, la membrana se colocó en una dilución 1/1000 de suero hiperinmune de llama inmunizado con SARS-CoV-2 diluido en tampón de bloqueo, durante una hora a 60 rpm. La membrana se retiró de la dilución de suero y se colocó en la solución de lavado (1X PBS, Tween 20) durante 5 minutos a 60 rpm; este proceso de lavado se realizó tres veces. La membrana se colocó en una dilución 1/5000 de anticuerpo anti-IgG de llama conjugada con peroxidasa diluida en tampón de bloqueo durante una hora a 60 rpm. La membrana se colocó sobre una base de mica de plástico transparente y se añadió la solución de detección (Western blot ultrasensible HRP sustrato-TAKARA) preparada según las recomendaciones del fabricante y se detectó la señal quimio luminiscente utilizando el sistema de detección Chemidoc XRS+ (BioRad).

*Prueba de neutralización viral de reducción de placa*

Los niveles de anticuerpos neutralizantes son medidos mediante la PRNT, que es considerada la prueba estándar de oro para medir los anticuerpos neutralizantes de un suero ^15^. Se sembraron células Vero 81 (1,8 × 105 células por pocillo) en placas de cultivo celular de 24 pocillos en medio EMEM suplementado con FBS al 10% y se incubaron durante 24 horas a 37 °C. Las muestras de suero de llama se inactivaron con calor a 56 °C durante 30 minutos y diluciones en serie se incubaron con stock de virus SARS-CoV-2 (40-50 UFP) durante una hora a 37 °C. La mezcla de virus-suero y los controles (virus de control, medio de dilución) se inocularon en células Vero 81 y se incubaron a 37 °C durante 1 hora 30 minutos, y luego se agregó 1 mL de medio de recubrimiento de carboximetilcelulosa al 0,75%. Las células se incubaron a 37 °C en CO_2_ al 5% durante cinco días. Las células se tiñeron con cristal violeta y se cuantificó la UFP para el cálculo del título PRNT50. Cada muestra de suero se analizó por duplicado.

### Análisis estadístico

Se utilizó el software estadístico R Studio para calcular el título PRNT50 por análisis probit con intervalo de confianza (IC) del 95% ^15^. Para el análisis de comparación entre ensayos ELISA y PRNT se utilizó el programa Microsoft Excel 2016.

### Aspectos éticos

El estudio fue aprobado por el Comité de Ética Institucional para el Uso de Animales de Experimentación del Instituto Nacional de Salud del Perú (Código OI-040-20).

## RESULTADOS

El cultivo del virus SARS-CoV-2 tenía un título viral de 1,5 x 10^6^ UFP/mL, y la purificación del virus por precipitación dio como resultado una suspensión opaca después de resuspender el precipitado. La inactivación de los viriones se realizó calentando la muestra a 56 °C durante una hora, y se verificó por cultivo (datos no mostrados); y esta purificación se verificó analizando los viriones por RT-PCR; observándose la detección de los genes RdRP y S específicos que confirman la identidad del virus SARS-CoV-2 (Figura 1B). El secuenciamiento genómico determinó que el linaje genético del virus cultivado fue el linaje B.1.1.

En el análisis de electroforesis SDS-PAGE se observó que, el sobrenadante del cultivo del virus SARS-CoV-2; así como el medio de cultivo solo, contiene otras proteínas abundantes además de las proteínas del virión. Tras la purificación de los viriones por precipitación, estas proteínas del medio de cultivo son eliminadas y sólo se observaron las proteínas del virus SARS-CoV-2 (Figura 1B).

El ensayo ELISA para detectar anticuerpos contra el virus SARS-CoV-2 en suero de llama reveló que los títulos de anticuerpos IgG aumentaron en las muestras de suero de acuerdo con el aumento de los refuerzos de inmunización; y este aumento en la respuesta de anticuerpos fue más evidente a partir de la cuarta semana después del inicio de las inmunizaciones, alcanzando el máximo en el séptimo refuerzo (Figura 2A). Los resultados del análisis de Western Blot demostraron la reactividad del antígeno total del virus SARS-CoV-2 al suero inmune de llama obtenido después del séptimo refuerzo de inmunización con el mismo virus SARS-CoV-2; además no se observó reacción de los antígenos del virus con el suero de una persona sana (control negativo) (Figura 2B). La respuesta a la inmunización, evaluada por ensayos ELISA y PRNT, mostró un aumento progresivo de anticuerpos contra el virus SARS-CoV-2 a medida que aumentaban las inmunizaciones (Figuras 2-5). Se observó un aumento en la neutralización del virus por el suero hiperinmune obtenido después del mayor número de inmunizaciones; evidenciado por la ausencia o reducción del conteo de placas virales en el ensayo de neutralización PRNT para los sueros recolectados después de la cuarta semana de inmunización (Figura 3).

**Figura 2:** Respuesta de anticuerpos de llama a la inmunización del virus SARS-CoV-2. Los anticuerpos contra el virus SARS-CoV-2 se midieron mediante ensayo ELISA en suero de llama después del refuerzo **(A).** Los viriones de SARS-CoV-2 purificados e inactivados se usaron como antígeno para el ensayo ELISA. El análisis de Western Blot **(B y C)** usó el lisado de proteína de virus completo (carriles 1 y 2), conjugado de IgG anti-llama con peroxidasa y BSA como proteína negativa (carril N). **(B)** Suero hiperinmune de llama como anticuerpo primario. **(C)** Suero humano negativo a SARSCoV-2 como anticuerpo primario. (M) Marcador de peso molecular de proteínas (Thermo Scientific # 26623).

**Figura 3:** Prueba de Neutralización por Reducción de Placa (PRNT) de suero hiperinmune de llama. CN: Control negativo (solo células, sin virus), CP: Control de virus.

La potencia o capacidad de neutralización viral del suero de llama se mide por los valores PRNT50 del ensayo PRNT, y se observa que estos valores son mayores a medida que se aumentan las dosis de inmunización y se correlacionan con los títulos de anticuerpos de los sueros estudiados (Figura 4-5). El valor PRNT50 máximo alcanzado después de los siete refuerzos fue PRNT50 = 6506,3 (IC al 95 %: 4202,7-11414,2).

**Figura 4:** Análisis de los resultados de la Prueba de Neutralización por Reducción de Placas (PRNT). El valor de PRNT50 se calculó a partir de los recuentos de reducción de placas y se indica dicho valor luego de cada refuerzo de inmunización (Refuerzos 3 al 7).

**Figura 5:** Análisis de comparación entre ensayos ELISA y PRNT.

## DISCUSIÓN

El presente estudio analizó la respuesta inmune de la llama a la inmunización con el virus SARS-CoV-2 (linaje B.1.1), y la capacidad de este suero hiperinmune para neutralizar la infección por el virus (linaje B.1.1) en un cultivo de células Vero; mostrando resultados alentadores en la investigación de nuevos posibles tratamientos de COVID-19 en base a nanoanticuerpos de camélidos. En nuestro estudio se encontró que después de la cuarta semana hubo un aumento significativo en la serorreactividad al virus SARS-CoV-2 (linaje B.1.1), con títulos más altos de anticuerpos en el séptimo refuerzo de inmunización u octava semana. Asimismo, el ensayo PRNT mostró una neutralización viral visible que fue concordante con los resultados de ELISA y Western Blot analizados. Esto sugiere que el suero hiperinmune de llama podría ser usado como una posible fuente de anticuerpos terapéuticos contra las infecciones por el virus SARS-CoV-2.

Actualmente, no existe un tratamiento claramente definido para combatir la infección por el virus SARS-CoV-2, en casos de COVID-19, aunque se han realizado estudios de anticuerpos capaces de neutralizar o inhibir la infección por el virus ^16^^,^^17^, y la estrategia de control más eficaz en este momento es la vacunación en todo el mundo. Sin embargo, la aparición continua de nuevas variantes genéticas del virus representa un desafío tanto a los tratamientos como a las vacunas existentes ^1^^,^^4^.

Algunas especies de camélidos como la llama (*Lama glama*) y el camello (*Camelus sp.*) son de especial interés en el estudio y producción de sueros neutralizantes de venenos ^18^^,^^19^. Estudios previos han descrito la capacidad del suero inmune de llama para neutralizar los venenos de algunas especies de serpientes^14^, y del suero de camelidos para neutralizar los venenos de escorpiones ^18^^,^^19^.

Los diferentes estudios con el virus SARS-CoV-2 requieren tener el virus en forma pura, ya sea en su forma activa para estudios de infección, o el virus inactivo para utilizarlo como antígeno, por ejemplo en inmunizaciones ^20^^,^^21^; para ello, los restos celulares y el componente del medio deben ser eliminados de los cultivos virales, para que no interfieran con los procedimientos experimentales. La mayoría de los protocolos para la concentración y purificación de virus utilizan un proceso de precipitación de viriones del cultivo, utilizando soluciones saturadas; en nuestro estudio, se utilizó una solución saturada de polietilenglicol y cloruro de sodio para precipitar viriones del sobrenadante del cultivo del virus SARS-COV-2 en células Vero, y este precipitado se resuspendió en tampón HEPES.

Los viriones purificados también deben ser inactivados para procedimientos experimentales que no impliquen infección por el virus o no lo requieran en su forma activa, para evitar el riesgo de infección del personal. Se han documentado diferentes métodos de inactivación como la inactivación por radiación gamma, luz UV-C, formaldehído, y glutaraldehído ^20^^-^^23^. En este estudio se evaluaron dos métodos de inactivación de virus, utilizando beta-propiolactona e inactivación por calor, ambos métodos resultaron efectivos, pero se optó por utilizar la inactivación por calor para evitar el riesgo de modificaciones químicas de los viriones, o toxicidad por beta-propiolactona; ya que estos viriones se utilizarían para las inmunizaciones en la llama.

El adyuvante más utilizado para la inmunización en muchos estudios es el adyuvante de Freund; en nuestro estudio utilizamos el adyuvante GERBU FAMA ^14^, que ha sido diseñado para la inmunización de animales mayores, para producir una mejor respuesta inmune y menos reacciones alérgicas o efectos adversos. Además, la inmunización subcutánea de cada dosis se utilizó en diferentes puntos de inoculación porque se ha reportado que produce una mejor respuesta inmune. El adyuvante GERBU FAMA produjo una buena respuesta inmunológica como lo demuestran los resultados de ELISA y Western Blot.

La respuesta inmune de la llama después de las inmunizaciones con el virus se evidenció en el aumento de los títulos de anticuerpos específicos para el virus SARS-CoV-2, a partir de la cuarta semana de inmunización, medidos por los ensayos ELISA y PRNT, utilizando los viriones inactivados de SARS-CoV-2 como antígeno. El análisis de Western Blot demostró el reconocimiento de las proteínas del antígeno total del virus SARS-CoV-2 por los anticuerpos totales contra el virus, presentes en el suero obtenido después del séptimo refuerzo de inmunización. Debido a que las inmunizaciones se realizaron con el virus SARS-CoV-2 completo purificado e inactivado; la respuesta inmune de la llama producirá anticuerpos contra las diferentes proteínas del virus; especialmente para las proteínas más inmunogénicas o abundantes; por esta razón, el ensayo Western Blot muestra el reconocimiento de varias proteínas virales por parte de los anticuerpos presentes en el suero hiperinmune.

Se observó la neutralización del linaje B.1.1 del virus SARS-CoV-2 por los sueros recolectados a partir de la cuarta semana de inmunización; observándose el aumento progresivo de los valores de neutralización PRNT50 a mayores dosis de inmunización, PRNT50 = 1:6506,3 (IC al 95%: 4202,7-11414,2). Entonces, nuestro suero de llama hiperinmune también podría servir como fuente para la purificación de anticuerpos específicos dirigidos a proteínas virales clave como *Spike* o RBD; o incluso para aislar anticuerpos monoclonales de la llama inmunizada.

En diferentes estudios destinados a producir anticuerpos específicos, se han inmunizado diferentes especies de animales, como musarañas , ratones transgénicos que expresan ACE2 humano, hamsters ^24^^,^^25^ y alpacas ^26^^,^^27^; pero estas inmunizaciones generalmente se realizan con antígenos puros o proteínas recombinantes como la proteína espiga. En nuestro estudio; de forma similar a otros estudios en camélidos con el SARS-CoV-2 ^26^^,^^27^, la inmunización de una llama con el virus SARS-CoV-2 completo ha demostrado generar anticuerpos que pueden reconocer los antígenos del virus SARS-CoV-2 (linaje B.1.1) y neutralizar la infección en células Vero.

Inicialmente, al no existir tratamientos para una nueva enfermedad como la COVID-19, se planteó como posible terapia el uso de plasma inmune de personas que habían superado la infección por COVID-19, obteniendo resultados a favor y en contra de estudios preliminares ^8^^-^^10^; posteriormente, se han aislado anticuerpos monoclonales de la sangre de pacientes infectados que muestran actividad neutralizante a la infección por el virus SARS-CoV-2 ^28^^,^^29^; algunos de los cuales, como REGN-COV2, un cóctel de mAb ha sido aprobado por la Administración de Alimentos y Medicamentos de los Estados Unidos (FDA, por sus siglas en inglés) para el tratamiento de COVID-19 ^30^; pero también algunos de estos medicamentos basados en anticuerpos monoclonales han ido perdiendo eficacia por la aparición de variantes genéticas del virus SARS-CoV-2 (Alfa, Beta, Gama, Delta y Ómicron). Actualmente, ya existen medicamentos aprobados por la FDA orientados a tratar personas vulnerables con casos leves o moderados de la COVID-19 (remdesivir, paxlovid y lagevrio, entre otros) ^30^.

El suero hiperinmune completo de llama contiene un gran número de anticuerpos, de los cuales sólo una fracción de ellos son anticuerpos neutralizantes; por ello, la utilidad del suero inmune con actividad neutralizante del virus SARS-CoV-2 radica en el potencial para identificar y aislar anticuerpos que neutralicen dianas específicas del virus, como la proteína espiga y su dominio RBD; que es la proteína que se une al receptor de la célula ACE2 y permite la infección por el virus SARS-CoV-2. En nuestro estudio, el suero hiperinmune de llama reaccionó a las bandas de proteínas del virus SARS-CoV-2 completo en el análisis de Western Blot; lo que sugiere que las inmunizaciones con el virus completo indujeron también anticuerpos contra la proteína de espiga en el suero; esto podría explicar en parte la capacidad del suero inmune para neutralizar el virus SARS-CoV-2 en cultivo de células Vero; ya que la proteína espiga es clave para la infección por el virus en las células.

Algunas limitaciones del estudio fueron trabajar con anticuerpos totales y no poder purificar o aislar los anticuerpos neutralizantes específicos del virus SARS-CoV-2. Además, en este estudio no se pudieron incluir más variantes del virus SARS-CoV-2 debido a la complejidad de su cultivo en un ambiente de bioseguridad tipo 3 y de estandarizar las pruebas de neutralización en cultivos celulares con cada variante del virus. Por último, solo se identificó al virus SARS-CoV-2 (linaje B.1.1), por lo tanto, futuros estudios son requeridos para evidenciar si el efecto encontrado en el presente estudio podría observarse en otros linajes o variantes.

En conclusión, nuestro estudio demostró que la inmunización de una llama (*Lama glama*) con el virus SARS-CoV-2 (linaje B.1.1) produce una respuesta inmune específica de anticuerpos y, por lo tanto, los anticuerpos en el suero hiperinmune tienen una actividad neutralizante viral en el cultivo de células Vero. Nuestros hallazgos revelan que las llamas podrían ser animales útiles para la evaluación terapéutica de anticuerpos contra la infección por el virus SARS-CoV-2 (linaje B.1.1).
